# Phenotypes of Jackhammer esophagus in patients with typical symptoms of gastroesophageal reflux disease responsive to proton pump inhibitors

**DOI:** 10.1038/s41598-018-27756-9

**Published:** 2018-07-02

**Authors:** Ivan Kristo, Katrin Schwameis, Svenja Maschke, Alexander Kainz, Erwin Rieder, Matthias Paireder, Gerd Jomrich, Sebastian F. Schoppmann

**Affiliations:** 1 Department of Surgery, Upper GI Research & Service, Medical University of Vienna, Vienna, Austria; 2 Department of Internal Medicine III, Medical University of Vienna, Vienna, Austria

## Abstract

This trial was designed to assess the prevalence and characteristics of Jackhammer esophagus (JE), a novel hypercontractile disorder associated with progression to achalasia and limited outcomes following anti-reflux surgery in patients with typical symptoms of GERD and responsiveness to proton pump inhibitor (PPI) therapy. Consecutive patients, who were referred for surgical therapy because of PPI responsive typical symptoms of GERD, were prospectively assessed between January 2014 and May 2017. Patients diagnosed with JE subsequently underwent rigorous clinical screening including esophagogastroduodenoscopy (EGD), ambulatory pH impedance monitoring off PPI and a PPI trial. Out of 2443 evaluated patients, 37 (1.5%) subjects with a median age of 56.3 (51.6; 65) years were diagnosed with JE and left for final analysis. Extensive testing resulted in 16 (43.2%) GERD positive patients and 5 (13.9%) participants were observed to have an acid hypersensitive esophagus. There were no clinical parameters that differentiated phenotypes of JE. The prevalence of JE in patients with typical symptoms of GERD and response to PPI therapy is low. True GERD was diagnosed in less than half of this selected cohort, indicating the need for objective testing to stratify phenotypes of JE. (NCT03347903)

## Introduction

Gastroesophageal reflux disease (GERD) is suggested to be involved in the induction of a novel hypercontractile condition that is not observed in healthy volunteers and was nicknamed Jackhammer esophagus (JE)^1,2^. According to the Chicago Classification (CC) version 3.0, an algorithm classifying high-resolution manometry (HRM) studies, this new phenotype of hypercontractility was determined to be present in subjects where ≥20% of swallows exceeded a distal contractile integral (DCI) of >8000 mmHg-s-cm in context of normal contractile propagation^3^.

Worryingly, recent observations indicated a progressive nature of the disease in 25% of patients with further development to type II and III achalasia^4,5^. Furthermore, nutcracker esophagus, a phenotype of hypercontractility that, in contrast to JE, was also observed in healthy controls, was associated with adverse postoperative outcomes following laparoscopic anti-reflux surgery^6^. Conceptually, these findings irritate surgical considerations, as implementation of HRM as a mandatory preoperative examination in patients with high evidence of GERD is discussed controversially in literature^7–11^. As a consequence, there is a subgroup of patients, where despite of surgical excellence and specialization, we fail to achieve good outcomes.

Therefore, this trial was designed to assess the prevalence and characteristics of JE in patients presenting with typical symptoms of GERD and therapeutic PPI response, referred for surgical anti-reflux therapy at a specialized centre.

## Results

### Baseline data

Out of 2443 patients referred and evaluated within the study period, 41 (1.7%) subjects were diagnosed with JE. Four patients had to be excluded due to previous gastrointestinal surgery (n = 2) and denial of pH metry (n = 2), which left 37 (females n = 18, 48.6%) individuals for the final analysis. Patients included in this trial had a median age of 56.3 (51.6; 65) years with 54.1% (n = 20) presenting with hiatal hernia in EGD. Esophagitis was observed in 9 patients (24.3%), consisting of 2 (22.2%) participants with LA grade B, 4 (44.4%) individuals with LA grade C and 3 (33.3%) patients with LA grade D esophagitis. Histopathology revealed the diagnosis of Barrett’s esophagus in 3 (8.1%) patients. Further baseline data are delineated in Table 1.

**Table 1:** Baseline characteristics of patients with Jackhammer esophagus referred for surgical anti-reflux therapy.

	Jackhammer esophagus
Patients	37
Age (years)	56.3 (51.6; 65)
Sex	
*Female n(%)*	18 (48.6)
*Male n(%)*	19 (51.4)
BMI (kg/m^2^)	26.1 (23.1; 31)
**Endoscopy n(%)**	
Esophagitis	9 (24.3)
hiatal hernia	20 (54.1)
Barrett’s esophagus	3 (8.1)
**Habits n(%)**	
smoking	14 (37.8)
alcohol consumption	
*daily*	3 (8.1)
*frequently*	16 (43.2)
*never*	18 (48.6)

**Figure 1:**
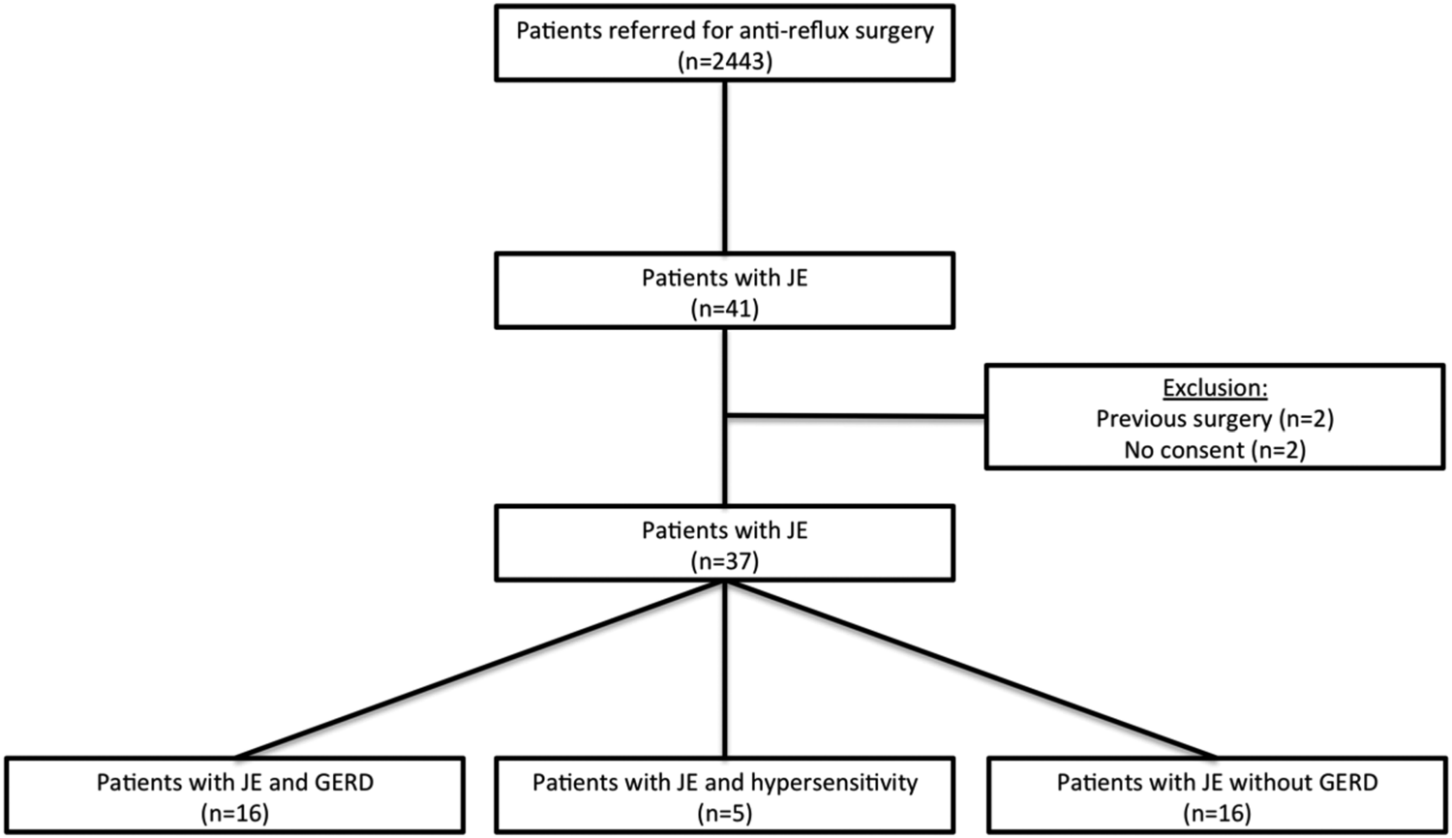
Study flow of patients with hypercontractile esophagus and involvement of gastroesophageal reflux disease and hypersensitivity JE…Jackhammer esophagus GERD…Gastroesophageal reflux disease

GERD was diagnosed in 16 (43.2%) patients, whereas acid hypersensitive esophagus was observed in 5 (13.9%) participants. Sixteen patients (43.2%) had no evidence of GERD (Fig. 1).

### Symptom assessment and PPI response

Patients reported heartburn (54.1%; n = 20) and dysphagia (51.4%; n = 19) as predominant symptoms with overall symptom duration of 2 (0.13; 9.5) years. Non-cardiac chest pain was noticed in 35.1% (n = 13), whereas regurgitations were observed in 18 (48.6%) patients (Table 2). All patients reported more than one symptom. Daily PPI intake was registered in 29 (78.4%) subjects. Patients experienced PPI mediated complete resolution of heartburn and or/regurgitation in 10% (n = 2) and partial resolution in 90% (n = 18). As far as typical JE symptoms are concerned, partial response to medical therapy was monitored in 6.9% (n = 2), whereas 93.1% (n = 27) described no effect of a PPI trial.

**Table 2:** Symptom presentation of patients with Jackhammer esophagus and symptoms of gastroesophageal reflux disease.

	Jackhammer esophagus
**Symptoms n(%)**	
heartburn	20 (54.1)
dysphagia	19 (51.4)
*grade I* + *II*	18 (48.6)
*grade III*	8 (21.6)
chest pain	13 (35.1)
regurgitation	18 (48.6)
globus sensation	7 (18.9)
coughing	8 (21.6)
hoarseness	10 (27)
symptom duration (years)	2 (0.13; 9.5)

### Subgroup analysis

There were no differences concerning age (p = 0.445), sex (p = 0.337) or BMI (0.463) between JE-GERD_pos_, JE-GERD_neg_ and JE_Hyper_ patients. Furthermore, symptoms like heartburn (p = 0.167), regurgitation (p = 0.250), dysphagia (p = 0.714) or non-cardiac chest pain (p = 0.906) as well as their duration (p = 0.319) did not discriminate groups. Intake of PPI (p = 0.377) and effect on heartburn (p = 0.494) or Jackhammer specific symptoms (p = 0.287) were also similar.

JE-GERD_pos_ patients had significantly higher counts of total reflux episodes (p = 0.027), which was mainly credited to acidic reflux episodes (p = 0.022) and not to non-acidic (p = 0.852) or gas containing (p = 0.583) reflux. CBR was significantly impaired in patients with JE-GERD_pos_. Quality of life and HRM did not allow differentiation between groups. Descriptive data are provided in Table 3.

**Table 3:** Differences between patients with Jackhammer esophagus with/without gastroesophageal reflux disease or hypersensitive esophagus.

	JE-GERD_pos_	JE_Hyper_	JE-GERD_neg_	p-value
Patients	16 (43.2)	5 (13.5)	16 (43.2)	
Sex n (%)				NS
*female*	6 (37.5)	2 (40)	10 (62.5)	
*male*	10 (62.5)	3 (60)	6 (37.5)	
Age (years)	59 (50.3; 65.8)	52.1 (51.9; 53.6)	57.5 (51.6; 59.4)	NS
BMI (kg/m^2^)	26.4 (21.6; 32.6)	24.3 (23.2; 27)	26.1 (23.1; 28.7)	NS
**Symptoms n (%)**				
Heartburn	10 (62.5)	4 (80)	6 (37.5)	NS
Dysphagia	9 (56.3)	3 (60)	7 (43.8)	NS
Chest pain	6 (37.5)	2 (40)	5 (31.3)	NS
Regurgitation	8 (50)	4 (80)	6 (37.5)	NS
Impact dysphagia questionnaire	6 (1.5; 16)	15 (9; 15)	12 (1; 17)	
Symptom duration (years)	2 (0.3; 9)	10 (1; 20)	1 (0; 5)	NS
daily PPI intake n (%)	14 (87.5)	5 (100)	10 (62.5)	NS
PPI response (heartburn) n (%)				NS
*full*	1 (6.3)	1 (20)	0	
*partial*	15 (93.8)	4 (80)	16 (100)	
*no effect*	0	0	0	
PPI response (Jackhammer symptoms) n (%)				NS
*full*	0	0	0	
*partial*	1 (6.3)	1 (20)	0	
*no effect*	15 (93.8)	4 (80)	16 (100)	
**Endoscopy n (%)**				
hiatal hernia	12 (75)	2 (40)	6 (37.5)	NS
esophagitis	9 (56.3)	0	0	0.0001
Barrett’s esophagus	3 (18.8)	0	0	NS
**High-resolution manometry**				
mulitpeaked configuration n (%)	8 (50)	3 (60)	4 (25)	NS
Outflow obstruction n (%)	7 (43.8)	0	10 (62.5)	0.049
DCI_max_ (mmHg·sec·cm)	11729 (10619; 20973)	10413 (10287; 10878)	10871 (8877; 13494)	NS
DCI_Hyper_ (mmHg·sec·cm)	10070 (8999; 13695)	9336 (9088; 10390)	9004 (8519; 10881)	NS
Number of hypercontractile swallows	6 (4; 8.5)	7 (5; 10)	4.5 (3; 8)	NS
Peak peristaltic amplitude (mmHg)	262 (240; 320)	264 (215; 278)	295 (240; 317)	NS
Complete bolus rate (%)	95 (20; 100)	100 (100; 100)	100 (100; 100)	0.04
**ambulatory impedance pH monitoring**				
time pH < 4 (%)				
*total*	6.25 (4.7; 10.1)	3 (0.5; 3.4)	0.8 (0.4; 1.9)	0.0001
*recumbent*	4 (0; 6)	0	0 (0; 0.3)	0.019
*upright*	10 (5.1; 16.2)	4 (0.9; 6.1)	1.35 (0.5; 2.9)	0.001
*postprandial*	8.4 (5; 16.5)	4.1 (0.6; 5)	1.3 (0.5; 2.3)	0.0001
Reflux episodes				
*Total*	45.5 (24; 65.5)	41 (38; 42)	17 (8; 30)	0.027
*Acidic*	41 (13.5; 51)	27 (17; 28)	7 (4; 18)	0.022
*Non-acidic*	9 (6; 20)	11 (7; 14)	10 (5; 15)	NS
*Gas*	6 (3; 10)	9 (6; 10)	5 (2; 11)	NS
**Quality of Life**				
GERD-HRQL (on PPI)	11 (6; 21)	11 (6; 17)	11 (1; 21)	NS
GERD-HRQL (off PPI)	13 (9;18)	14 (13; 22)	13 (3; 21)	NS

JE-GERD_pos_ Patients with Jackhammer esophagus and gastroesophageal reflux disease.

JE_Hyper_ Patients with Jackhammer esophagus and hypersensitive esophagus.

JE-GERD_neg_ Patients with Jackhammer esophagus without gastroesophageal reflux disease.

DCI_max_ Distal contractile integral of maximum hypercontractile swallow.

DCI_Hyper_ Distal contractile integral of all hypercontractile swallows.

GERD-HRQL Gastroesophageal Reflux Disease – Health Related Quality of Life.

## Discussion

This is the first study prospectively assessing the prevalence of JE in a selected cohort of patients presumptive of GERD. We could demonstrate that JE was found in 1.7% of patients referred for surgical therapy at a specialized academic centre. Clinical presentation and response to PPI was insufficient to stratify phenotypes of JE. Acidic reflux episodes discriminated JE and GERD supporting the thesis of an acidic reflux induced phenotype of hypercontractility.

The introduction of HRM studies revisited the concept of esophageal hypercontractility and led to the introduction of a novel hypercontractile disorder, which in contrast to nutcracker esophagus was never observed in healthy controls^1^. Interestingly, JE, named after its common multi-peaked appearance, was registered in 1.7% of patients with typical symptoms of GERD responsive to PPI therapy, which goes in line with recent observations, where JE was found in up to 5% of unselected patients undergoing HRM^12,13^.

From a pathophysiological point of view, affected patients seem to represent a heterogeneous population with demanding therapeutic implications. First and foremost, outflow obstruction may mediate hypercontractility. Burton *et al*. reported that overfilling of a laparoscopic adjustable gastric band resulted in hypercontractile and repetitive esophageal contractions, which was also observed in another study, when functional obstruction was present^14,15^. In our population, 45.9% of subjects where diagnosed with concomitant outflow obstruction, significantly more frequent in GERD negative patients, discriminating a distinct phenotype of disease. As confirmation, Jia *et al*. observed similar outflow obstruction rates results in their cohort^13^. Secondly, a hyper-cholinergic state may lead to asynchrony between muscular layers noticed frequently in hyerpcontractility that can be reversed by atropine^16,17^.

Moreover, GERD is suggested to play a major role in induction of hypercontractility. Early acid perfusion studies have demonstrated that acid was able to provoke spasms, motility changes and the perception of non-cardiac chest pain, a symptom frequently observed in JE^18,19^. Unfortunately, there is hardly any data focusing on GERD in JE. Mallet *et al*. observed that prevalence of GERD was higher in JE when compared to the general population^12^. Nevertheless, endoscopy and ambulatory pH monitoring, cornerstones in the diagnosis of GERD, were not performed in nearly a third of their patients. Additionally, ambulatory pH monitoring was used without impedance, which limits diagnostic yield^20^.

Importantly, GERD was only diagnosed in 43.2% of patients with JE all presumptive of GERD. Although typical symptoms of heartburn and regurgitation are most reliable for making a presumptive diagnosis of GERD based on history alone and are endorsed by societies as a pragmatic approach in daily clinical practice, modern recommendations include empiric PPI trial and objective testing to substantiate diagnosis of true GERD^11,21,22^.

We could observe that total reflux episodes were higher in JE-GERD_pos_ patients, which was based on more acidic reflux episodes. As far as gas reflux and non-acidic reflux is concerned, there were no differences between groups. Furthermore, JE-GERD_pos_ patients were associated with impaired complete bolus rates. Therefore, our data support the pathophysiological concept of JE induction by reflux of acidic gastric contents.

Acid-hypersensitive esophagus was remarked in 13.5% of our cohort. Thus, our data acknowledge that functional esophageal disorders are associated with visceral hypersensitivity and are not always a result of GERD^23^. Mujica *et al*. recognized that symptoms in patients with nutcracker esophagus could be reproduced by stepwise balloon distensions emphasizing a lower threshold for visceral sensitivity^24^. This may explain the fact that 43.2% of our patients reported typical PPI responsive symptoms without evidence of GERD.

Therapeutic aspects of GERD in JE are complex. Recently, a longitudinal study revealed that 25% of patients with JE progressed to type III achalasia within 2 years, whereas progression to type II achalasia was also observed^4,5^. Potentially, these individuals may profit from modern peroral endoscopic myotomy (POEM) to overcome obstructed outflow and were represented in our JE-GERD_neg_ subgroup^25^.

Currently, it is unknown if control of GERD will limit development to achalasia, improve JE or if JE itself is responsible for a proportion of adverse postoperative outcomes at specialized centres. Data on surgical or medical therapy for GERD in JE are more than scarce. Crespin *et al*. reported on two patients with JE that underwent laparoscopic Nissen fundoplication (LNF) and returned to regular esophageal motility^26^. Furthermore, Roman *et al*. followed 12 patients with JE that received PPI or LNF for control of GERD and realized that 7 of them improved significantly^2^. In contrast, PPI failed to achieve symptom control in a randomized placebo controlled trial in nutcracker esophagus, a hypercontractile condition that was similar in treatment response to JE in a small uncontrolled trial^27,28^. Furthermore, Winslow *et al*. reported that patients with spastic motor disorders had higher rates of persisting GERD symptoms and postoperative dysphagia after laparoscopic anti-reflux surgery^6^. Nevertheless, preoperative manometry, which is necessary to detect JE in this selected cohort is discussed controversially in literature. Whereas gastroenterological societies and an expert advisory panel recommend preoperative manometry mainly to exclude conditions like achalasia or distal esophageal spasm, large surgical societies still question the support in literature for its mandatory use^7,9–11^. Frantzides *et al*. reported a series of 628 consecutive laparoscopic floppy Nissen procedures, where manometry was either used as routine evaluation or selective in presence of dysphagia, odynophagia or abnormal motility on videofluoroscopy^29^. The authors stated that selective use of manometry was cost effective and safe, as it did not increase rates of postoperative dysphagia even if dysmotility was diagnosed. Following this concept nearly half of our cohort would miss the diagnosis of JE, as they did not experience dysphagia. Furthermore, prospective randomized data of preoperative and postoperative use of manometry failed to affect or detect postoperative surgical outcomes^30,31^. Given that we could show that JE was rather low in our cohort, these conflicting statements may just be a result of underpowered studies.

Limitations of this study have also to be addressed. Although this is the largest study prospectively evaluating patients with JE, single centre design and low prevalence restrict the number of patients with disease. Furthermore, we emphasize that the Chicago classification uses JE as a term for a heterogeneous population with varying pathophysiological background that may influence symptoms and reflux burden. Therefore, our results and conclusions have to be interpreted with care. Strengths represent the prospective assessment of data that limits potential selection bias and the rigorous examination of patients by diagnostic cornerstones of GERD.

In conclusion, this study shows that the prevalence of JE in patients with typical symptoms of GERD and empirical response to PPI therapy is low. True GERD was diagnosed in less than half of this selected cohort, indicating the need for additional objective testing to stratify phenotypes of JE. Mandatory preoperative HRM should be recommended by surgical societies to determine presence of hypercontractile disorders that potentially limit postoperative outcomes.

## Methods

### Patient selection

All consecutive patients, who were referred for surgical therapy because of PPI responsive typical symptoms of GERD (heartburn and/or regurgitation) between January 2014 and May 2017, were included in this trial and underwent HRM at our tertiary academic referral centre for gastrointestinal research and service. Patients showing ≥20% of swallows with DCI >8000 mmHg-s-cm in context of normal contractile propagation^3^ were analysed. Exclusion criteria were presence of achalasia, distal esophageal spasm, history of previous gastrointestinal surgery, mechanical esophageal obstruction or other diseases explaining their symptoms (i.e. eosinophilic esophagitis, systemic sclerosis).

### Study flow

After inclusion, subjects underwent esophagogastroduodenoscopy (EGD) off PPI to determine presence of GERD, classified according to the Los Angeles (LA) classification^32^. Medical history, symptoms and Impact Dysphagia Questionnaire (IDQ) were assessed in a face-to-face interview with study participants being at least 14 days off PPI. Dysphagia and non-cardiac chest pain were graded utilizing a standardized assessment according to Mellow and Pinkas^33^. Response to PPI therapy was tested by an 8-week double-dose course of esomeprazole therapy to differentiate between improvement of typical (heartburn, regurgitation) and/or Jackhammer specific symptoms (dysphagia, non-cardiac chest pain). Partial response to PPI therapy was defined as any reduction of symptom intensity, whereas full response to PPI therapy resulted in resolution of symptoms. For subgroup analysis patients were divided in GERD positive (JE-GERD_pos_), GERD-negative (JE-GERD_neg_) and patients with acid hypersensitive esophagus (JE_Hyper_).

Quality of life was determined using the Gastroesophageal Reflux Disease-Health Related Quality of Life (GERD-HRQL) on (GERD-HRQL/+PPI) and at least 8 weeks off PPI therapy (GERD-HRQL/−PPI). Concluding, 24 h impedance pH metry was performed in all patients. All patients underwent informed consent and the institutional review board of the Medical University of Vienna approved the study protocol. All methods were performed in accordance with relevant guidelines and regulations.

### High-resolution impedance manometry

High-resolution impedance manometry was performed using a solid-state catheter with 32 circumferential pressure transducers at 1 cm intervals and dual impedance sensors (Sandhill Scientific, Highlands Ranch, CO, USA) as previously described^34,35^. In brief, the catheter was inserted transnasally and positioned to record pressure from hypopharynx to the stomach in supine position. Esophageal body motility was assessed with 10 liquid swallows of 5 ml at 30-second intervals. BioView™ (Sandhill Scientific, Highlands Ranch, CO, USA) was utilized to interpret data according to the CC v3.0^3^. The DCI was calculated from the proximal pressure trough to the proximal level of the LES integrating amplitude, duration and length of contraction exceeding 20 mmHg. In addition to regular CC specific metrics all hypercontractile swallows were evaluated. Complete bolus transit was defined as described by Tutuian *et al*. with complete bolus rate (CBR) being the percentage of complete bolus transit referred to all liquid swallows^36^.

### Ambulatory 24-hour pH impedance monitoring

Patients had to stop PPI or H2 blocker intake at least 10 days before monitoring. A catheter containing impedance tracers and electrodes with internal reference for pH measuring was utilized (ComforTec ZAN-44; Sandhill Scientific, Highlands Ranch, CO, USA). Electrodes were positioned after locating the lower esophageal sphincter (LES) by HRM as previously described^37^. Patients were instructed to stick to their daily routine and enter symptoms, body position and meals. GERD was defined as the percentage of endoluminal pH < 4 exceeding 4.2% in the distal esophagus and/or endoscopic visible lesions according to the LA classification. An acid-hypersensitive esophagus was registered when normal esophageal acid exposure was associated with a symptomatic index greater than 50% and/or a symptom association probability (SAP) greater than 95%.

### Statistical analysis

Demographics are presented as mean with standard deviation if normally distributed or as median with interquartile ranges otherwise. Categorical variables are displayed as absolute numbers and percentages. High-resolution impedance manometry metrics are delineated as median with interquartile ranges. Chi-square or Wilcoxon rank test were applied as appropriate for comparison between groups. The Kruskal-Wallis method was applied for comparison between three groups. P-values ≤ 0.05 were considered statistically significant. Analyses were done using SPSS for Macintosh Version 24.0 (IBM Corp., Armonk, NY, USA). The dataset analyzed during the current study are available from the corresponding author on request.
